# Genomic selection using regularized linear regression models: ridge regression, lasso, elastic net and their extensions

**DOI:** 10.1186/1753-6561-6-S2-S10

**Published:** 2012-05-21

**Authors:** Joseph O Ogutu, Torben Schulz-Streeck, Hans-Peter Piepho

**Affiliations:** 1 Bioinformatics Unit, Institute for Crop Science, University of Hohenheim, Fruwirthstrasse 23, 70599 Stuttgart, Germany

## Abstract

**Background:**

Genomic selection (GS) is emerging as an efficient and cost-effective method for estimating breeding values using molecular markers distributed over the entire genome. In essence, it involves estimating the simultaneous effects of all genes or chromosomal segments and combining the estimates to predict the total genomic breeding value (GEBV). Accurate prediction of GEBVs is a central and recurring challenge in plant and animal breeding. The existence of a bewildering array of approaches for predicting breeding values using markers underscores the importance of identifying approaches able to efficiently and accurately predict breeding values. Here, we comparatively evaluate the predictive performance of six regularized linear regression methods-- ridge regression, ridge regression BLUP, lasso, adaptive lasso, elastic net and adaptive elastic net-- for predicting GEBV using dense SNP markers.

**Methods:**

We predicted GEBVs for a quantitative trait using a dataset on 3000 progenies of 20 sires and 200 dams and an accompanying genome consisting of five chromosomes with 9990 biallelic SNP-marker loci simulated for the QTL-MAS 2011 workshop. We applied all the six methods that use penalty-based (regularization) shrinkage to handle datasets with far more predictors than observations. The lasso, elastic net and their adaptive extensions further possess the desirable property that they simultaneously select relevant predictive markers and optimally estimate their effects. The regression models were trained with a subset of 2000 phenotyped and genotyped individuals and used to predict GEBVs for the remaining 1000 progenies without phenotypes. Predictive accuracy was assessed using the root mean squared error, the Pearson correlation between predicted GEBVs and (1) the true genomic value (TGV), (2) the true breeding value (TBV) and (3) the simulated phenotypic values based on fivefold cross-validation (CV).

**Results:**

The elastic net, lasso, adaptive lasso and the adaptive elastic net all had similar accuracies but outperformed ridge regression and ridge regression BLUP in terms of the Pearson correlation between predicted GEBVs and the true genomic value as well as the root mean squared error. The performance of RR-BLUP was also somewhat better than that of ridge regression. This pattern was replicated by the Pearson correlation between predicted GEBVs and the true breeding values (TBV) and the root mean squared error calculated with respect to TBV, except that accuracy was lower for all models, most especially for the adaptive elastic net. The correlation between the predicted GEBV and simulated phenotypic values based on the fivefold CV also revealed a similar pattern except that the adaptive elastic net had lower accuracy than both the ridge regression methods.

**Conclusions:**

All the six models had relatively high prediction accuracies for the simulated data set. Accuracy was higher for the lasso type methods than for ridge regression and ridge regression BLUP.

## Introduction

Genomic selection (GS), the prediction of genomic breeding values (GEBVs) using dense molecular markers, is rapidly emerging as a key component of efficient and cost-effective breeding programs. The prediction of GEBVs is currently undertaken using multiple methods with varying degrees of complexity, computational efficiency and predictive accuracy. Comparative evaluation of the performance of the existing methods is thus essential to identify those best suited to GS and determine when their performance is optimal. Here, we evaluate the relative performance of six regularized (penalized) linear regression models for GS. The methods comprise ridge regression (RR) [1], ridge regression best linear unbiased prediction (RR-BLUP) [2], the least absolute shrinkage and selection operator (lasso) [3-5], elastic net [6-8], adaptive lasso [9] and adaptive elastic net (ADAENET) [10]. The appeal and success of regularization models in many application domains, including genomic selection, relate to their use of penalties that facilitate fitting models with predictors that run into thousands, including many irrelevant to the response, far exceed the sample size, or are highly correlated, with high efficiency and prediction accuracy.

## Methods

### Data

An outbred population of 1000 individuals was simulated over 1000 generations, followed by 150 individuals over 30 generations, using the LDSO software [11]. Biallelic SNP markers (*n *= 9990) were distributed on 5 chromosomes, each 1 Morgan in size, at every 0.05 cM for a total of 1998 SNPs. For analysis, data corresponding to the last generation of the simulated pedigree of 20 sires, each mated to 10 different dams and yielding 15 progenies per dam were selected, for a total of 3000 progenies. For each full-sib family of 15 progenies, 10 progenies were genotyped and phenotyped (*n *= 2000 progenies) whereas the remaining 5 were genotyped but not phenotyped (*n *= 1000 progenies). The 3000 progenies served as the candidates for genomic prediction in this study. Our aim here is to predict (1) the true expectation of the phenotypes of the 1000 non-phenotyped candidates (i.e. the true genomic value, TGV) and (2) the true expectation of the phenotypes of the progenies of the 1000 non-phenotyped candidates (i.e., the true breeding value, TBV).

The marker information was stored in a matrix $X=\left\{x_{ij}\right\}$, with $x_{ij}$ denoting the marker covariate for the *i*-th genotype (*i *= 1, 2,..., *n*) and the *j*-th marker (*j *= 1, 2,..., *p*) for the biallelic SNP markers with alleles *A*_1 _and *A*_2 _coded as 1 for *A*_1_*A*_1_, -1 for *A*_2 _*A*_2 _and 0 for *A*_1 _*A*_2 _or *A*_2 _*A*_1_.

### The regularization models

The basic linear regression model used to predict GEBVs with all the six regularization models is:

(1) $$y=\mu 1_{n}+X\beta +e,$$

where **y**=(y_1_,...,y*_n_*)*^T ^*is the vector of observed phenotypes, **1***_n _*is a column vector of *n *ones and *μ *is a common intercept, **X **is a *n *× *p *matrix of markers; **β **is the vector of the regression coefficients of the markers and **e **is the vector of the residual errors with $\text{var}\left(e\right)=I\sigma_{e}^{2}$. In what follows, we assume that the observed phenotypes have been mean-centered.

### Ridge regression

Ridge regression [1] is ideal if there are many predictors, all with non-zero coefficients and drawn from a normal distribution [12]. In particular, it performs well with many predictors each having small effect and prevents coefficients of linear regression models with many correlated variables from being poorly determined and exhibiting high variance. RR shrinks the coefficients of correlated predictors equally towards zero. So, for example, given *k *identical predictors, each would get identical coefficients equal to 1/*k*th the size that any one predictor would get if fit singly [12]. RR thus does not force coefficients to vanish and hence cannot select a model with only the most relevant and predictive subset of predictors.

The ridge regression estimator solves the regression problem in (1) using ℓ_2 _penalized least squares:

(2) $$\hat{\beta }\left(\text{ridge}\right)=\underset{\beta }{\text{arg}\text{min}}{∥y-X\beta ∥}_{2}^{2}+\lambda {∥\beta ∥}_{2}^{2}$$

where ${∥y-X\beta ∥}_{2}^{2}=\sum_{i=1}^{n}{\left(y_{i}-x_{i}^{T}\beta \right)}^{2}$ is the ℓ_2 _-norm (quadratic) loss function (i.e. residual sum of squares), $x_{i}^{T}$ is the *i*-th row of **X**, ${∥\beta ∥}_{2}^{2}=\sum_{j=1}^{p}\beta_{j}^{2}$ is the ℓ_2 _-norm penalty on **β**, and λ ≥ 0 is the tuning (penalty, regularization, or complexity) parameter which regulates the strength of the penalty (linear shrinkage) by determining the relative importance of the data-dependent empirical error and the penalty term. The larger the value of λ, the greater is the amount of shrinkage. As the value of λ is dependent on the data it can be determined using data-driven methods, such as cross-validation. The intercept is assumed to be zero in (2) due to mean-centering of the phenotypes.

### Ridge regression BLUP

Ridge regression BLUP uses the same estimator as ridge regression but estimates the penalty parameter by REML as $\lambda =\sigma_{e}^{2}/\sigma_{\beta }^{2}$, where $\sigma_{e}^{2}$ is the residual variance, $\sigma_{\beta }^{2}$ is the variance of the regression coefficients and $\text{var}\left(\beta \right)=I\sigma_{\beta }^{2}$[2].

### Lasso

Lasso regression methods are widely used in domains with massive datasets, such as genomics, where efficient and fast algorithms are essential [12]. The lasso is, however, not robust to high correlations among predictors and will arbitrarily choose one and ignore the others and break down when all predictors are identical [12]. The lasso penalty expects many coefficients to be close to zero, and only a small subset to be larger (and nonzero). The lasso estimator [3] uses the ℓ_1 _penalized least squares criterion to obtain a sparse solution to the following optimization problem:

(3) $$\hat{\beta }\left(lasso\right)=\underset{\beta }{\text{arg}\text{min}}{∥y-X\beta ∥}_{2}^{2}+\lambda {∥\beta ∥}_{1},$$

where ${∥\beta ∥}_{1}=\sum_{j=1}^{p}\left|\beta_{j}\right|$ is the ℓ_1 _-norm penalty on **β**, which induces sparsity in the solution, and λ ≥ 0 is a tuning parameter.

The ℓ_1 _penalty enables the lasso to simultaneously regularize the least squares fit and shrinks some components of $\hat{\beta }\left(lasso\right)$ to zero for some suitably chosen *λ*. The cyclical coordinate descent algorithm [12] efficiently computes the entire lasso solution paths for *λ *for the lasso estimator and is faster than the well-known LARS algorithm [13]. These properties make the lasso an appealing and highly popular variable selection method. Even so, the lasso has three key shortcomings -- it lacks the oracle property (see below), is unstable with high-dimensional data and can not select more variables than the sample size before it saturates when *p *>*n *[9].

An oracle procedure can estimate the subset of true parameters with zero coefficients as exactly zero with probability tending to 1; that is, as well as if the true subset model were known beforehand [14]. An oracle estimator, furthermore, performs asymptotically consistent and efficient variable selection and produces asymptotically unbiased and normally distributed estimates of the nonzero coefficients [9,14]. The oracle property is closely related to the super-efficiency phenomenon [14]. However, the oracle property alone does not guarantee optimality of estimators. Optimal estimators must also satisfy certain additional and important regularity conditions besides having the oracle property, such as continuous shrinkage [9]. The lasso lacks the oracle property because it estimates the larger nonzero coefficients with asymptotically non-ignorable bias [14] and can only consistently perform variable selection when the predictor matrix (or the design matrix) satisfies a rather strong condition [9].

### Adaptive lasso

To remedy the problem of the lack of the oracle property, the adaptive lasso estimator was proposed [9]:

(4) $$\hat{\beta }\left(\text{AdaLasso}\right)=\underset{\beta }{\text{arg}\text{min}}{∥y-X\beta ∥}_{2}^{2}+\lambda \overset{p}{\underset{j=1}{\sum }}{\hat{\omega }}_{j}\left|\beta_{j}\right|,$$

where ${\hat{\omega }}_{j}$ (*j *= 1,..., *p*) are the adaptive data-driven weights, which can be estimated by ${\hat{\omega }}_{j}={\left(\left|{\hat{\beta }}_{j}^{ini}\right|\right)}^{-\gamma }$, where *γ *is a positive constant and ${\hat{\beta }}^{ini}$ is an initial consistent estimator of **β **obtained through least squares or ridge regression if multicolinearity is important [9]. The optimal value of *γ *>0 and λ can be simultaneously selected from a grid of values, with values of *γ *selected from {0.5, 1, 2}, using two-dimensional cross-validation [9]. The weights allow the adaptive lasso to apply different amounts of shrinkage to different coefficients and hence to more severely penalize coefficients with small values. The flexibility introduced by weighting each coefficient differently corrects for the undesirable tendency of the lasso to shrink large coefficients too much yet insufficiently shrink small coefficients by applying the same penalty to every regression coefficient [9]. For suitably chosen *λ*, the adaptive lasso performs as well as the oracle [9]. Despite being an oracle procedure, the adaptive lasso inherits the instability of the lasso for high-dimensional data.

### Elastic net

The elastic net (ENET) is an extension of the lasso that is robust to extreme correlations among the predictors [12]. To circumvent the instability of the lasso solution paths when predictors are highly correlated (e.g. SNPs in high linkage disequilibrium), the ENET was proposed for analyzing high dimensional data [6]. The ENET uses a mixture of the ℓ_1 _(lasso) and ℓ_2 _(ridge regression) penalties and can be formulated as:

(5) $$\hat{\beta }\left(\text{enet}\right)=\left(1+\frac{\lambda_{2}}{n}\right)\underset{}{\left(\left\{\underset{\beta }{\text{arg}\text{min}}{∥y-X\beta ∥}_{2}^{2}+\lambda_{2}{∥\beta ∥}_{2}^{2}+\lambda_{1}{∥\beta ∥}_{1}\right)\right\}},$$

On setting *α*=*λ*_2_/(*λ*_1_+*λ*_2_), the ENET estimator (5) is seen to be equivalent to the minimizer of:

(6) $$\hat{\beta }\left(\text{enet2}\right)=\underset{\beta }{\text{arg}\text{min}}{∥y-X\beta ∥}_{2}^{2},\;\text{subject}\;\text{to}\;P_{\alpha }\left(\beta \right)=\left(1-\alpha \right){∥\beta ∥}_{1}+\alpha {∥\beta ∥}_{2}^{2}\leq s\;\text{for}\;\text{some}\;s$$

where *P_α_*(**β**) is the ENET penalty [6]. The ENET simplifies to simple ridge regression when *α*=1 and to the lasso when *α*=0. The ℓ_1 _part of the ENET does automatic variable selection, while the ℓ_2 _part encourages grouped selection and stabilizes the solution paths with respect to random sampling, thereby improving prediction. By inducing a grouping effect during variable selection, such that a group of highly correlated variables tend to have coefficients of similar magnitude, the ENET can select groups of correlated features when the groups are not known in advance. Unlike the lasso, when *p *>>*n*, the elastic net selects more than *n *variables. Nonetheless, the elastic net lacks the oracle property.

### Adaptive elastic net

The adaptive elastic net is a mixture of the adaptive lasso and the elastic net that confers the oracle property to the elastic net and alleviates the instability of the adaptive lasso with high-dimensional data inherited from the lasso [6,9]. The adaptive lasso attains the oracle property whereas the elastic net fixes the multicolinearity problem. In essence, the ADAENET unites the ideas of the adaptively weighted ℓ_1 _penalty of the adaptive lasso and the elastic net regularization to confer the oracle property to the lasso and enhance its stability (selection consistency and asymptotic normality) with high-dimensional data by solving the optimization problem [10]:

(7) $$\hat{\beta }\left(\text{AdaEnet}\right)=\left(1+\frac{\lambda_{2}}{n}\right)\underset{}{\left(\left\{\underset{\beta }{\text{arg}\text{min}}{∥y-X\beta ∥}_{2}^{2}+\lambda_{2}{∥\beta ∥}_{2}^{2}+\lambda_{1}^{*}\overset{p}{\underset{j=1}{\sum }}{\hat{\omega }}_{\text{j}}\left|\beta_{j}\right|\right)\right\}},$$

where the elastic-net estimator $\hat{\beta }\left(\text{enet}\right)$ in (5) is first computed and then adaptive weights are constructed by:

$${\hat{\omega }}_{j}={\left(\left|{\hat{\beta }}_{j}\left(\text{enet}\right)\right|\right)}^{-\gamma },j=\text{ 1},\text{ 2},\ldots ,p,$$

and *γ *is a positive constant.

### Fitting and comparing models

The entire path of solutions (in λ) for the ridge regression, lasso and elastic net models were computed using the pathwise cyclical coordinate descent algorithms-- computationally efficient methods for solving these convex optimization problems-- in *glmnet *in R [12]. We used fivefold CV within *glmnet *to exclusively search for the optimal λ [12]. For the selected optimal λ we did another fivefold CV external to *glmnet *to determine the optimal value for *α*. A more computationally expensive two-dimensional CV may also be used to search for an optimal pair of (α, λ) over the two-dimensional grids of parameters for the ridge regression, lasso and the elastic net. The adaptive lasso was fit using the *parcor *package in R whereas the adaptive elastic net using an R function that calls the *elasticnet *kindly provided to us by Zou and Zhang. Values for each SNP marker were mean-centered except for RR-BLUP. For the adaptive elastic net, all markers with zero variance were excluded from analysis. Ridge regression BLUP was fit as a mixed model and estimated by REML. The fivefold CV external to *glmnet *used to select the optimal *α*, given the optimal *λ*, was also used to evaluate the prediction accuracies of all the six models. This particular CV entailed splitting the observations for each full-sib family into five parts, four of which were concatenated and used as the training set for estimating the regression coefficients for the markers, while the fifth served as a validation set. Pearson correlations between the simulated and predicted GEBVs were computed and used to evaluate predictive accuracy. Predictive accuracy was also assessed as the Pearson correlation between predicted GEBVs and (1) the true genomic value (TGV), (2) the true breeding value (TBV), as well as using the root mean squared error.

## Results and discussion

Predictive accuracy, expressed as the Pearson correlation between predicted GEBVs and the true genomic values (TGV) and the root mean squared error derived from TGV, ranked the elastic net, lasso and adaptive lasso above the adaptive elastic net, ridge regression and ridge regression BLUP (Table 1). A similar ranking was also produced based on the true breeding values (TBV) but with two notable differences. First, accuracy based on TBV was lower than that derived from TGV for all models, particularly for the adaptive elastic net, which ranked lower than the two ridge regression models. Second, root mean squared error was distinctly higher with respect to TBV than to TGV. The reduced accuracy reflects the fact that our models were trained to predict TGV and not TBV because the training data set consisted of phenotypes of the 2000 candidates and not phenotypes of their progenies. However, in most genomic selection studies, prediction accuracy is more often assessed relative to the true breeding value rather than relative to the true genomic value.

**Table 1:** Accuracy of predictions of the six models

Model	Pearson correlation					Root mean squared error	
	**5-fold cross-validation**			**TGV**	**TBV**	**TGV**	**TBV**

	**Mean**	**Min**	**Max**				

Elastic Net	0.5071	0.4486	0.5308	0.9233	0.8659	2.2276	3.4618

Lasso	0.5062	0.4466	0.5293	0.9240	0.8705	2.1642	3.5478

Adaptive Lasso	0.4951	0.4454	0.5152	0.9195	0.8759	2.0757	3.9911

RR	0.4717	0.4050	0.5037	0.8246	0.8213	2.9046	3.3767

RR-BLUP	0.4628	0.3905	0.4951	0.8455	0.8315	2.9894	3.6487

Adaptive Elastic Net	0.4285	0.4013	0.4667	0.8968	0.8112	2.3404	4.2325

Pearson correlation between GEBVs and (1) the observed values from the 5-fold cross-validation, (2) the true expectation of the phenotypes of the 1000 non-phenotyped candidates (TGV), (3) the true expectation of the phenotypes of the progenies of the 1000 non-phenotyped candidates (TBV); and the root mean squared error with respect to TGV and TBV.

The fivefold CV also ranked the models similarly to the correlations based on TGV and TBV. Based on CV, the elastic net and lasso performed better than ridge regression, ridge regression BLUP and the adaptive extensions of lasso and the elastic net. A previous study also found that the elastic net often outperforms RR and the lasso in terms of model selection consistency and prediction accuracy [6]. Thus, even though it possesses the oracle property and is robust to multicolinearity, and hence would be expected to have high predictive accuracy, the adaptive elastic net had lower accuracy than the other lasso type models in this study. This may suggest that the set of parameters selected for the elastic net were probably not optimal and hence the need for an efficient algorithm for selecting the three parameters for the adaptive elastic net simultaneously.

The RR and RR-BLUP penalties admit all markers into the model, resulting in a very large number of non-zero coefficients. The two ridge penalties shrink parameter estimates and will perform well for many markers with small effects but are less effective in forcing many predictors to vanish, as was the case for the data set simulated for the 2011 QTLMAS workshop, and cannot therefore produce parsimonious and interpretable models with only the relevant markers. All the models with the lasso penalty perform simultaneous automatic variable selection and shrinkage. The elastic net penalty provides a compromise between the lasso and ridge penalties and has the effect of averaging markers that are highly correlated and then entering the averaged marker into the model. Since they are numerous, the non-zero coefficients for the ridge regression are far smaller than the coefficients for the other methods.

If the number of markers (*p*) is more than that of phenotypes (*n*), the lasso will select at most *n *markers before it saturates, which may lower its predictive accuracy but some extensions of the lasso, such as the Bayesian lasso, alleviate this problem using marker-specific shrinkage [15]. The lasso tends to select only one of a set of highly correlated predictors and simply excludes all others in the group and hence cannot select two identical predictors as can the ENET [6]. However, both the lasso and ENET may sometimes fail to select the true model, but when the lasso can select the true model, the ENET also can [6].

The six methods we considered are closely related to many other regularized statistical learning procedures, many of which are also promising for GS. Examples of such models include boosted ridge regression [16], numerous lasso variations such as group lasso [17,18], which includes or excludes variables in groups, adaptive group lasso [19], lasso penalized generalized linear models [12], the Dantzig selector [20], a slightly modified version of the lasso, generalized elastic net [21]; smoothly clipped absolute deviation procedures that reduce bias and yield continuous solutions (SCAD) [14], reproducing kernel Hilbert spaces regression [22] and support vector regression [23]. Moreover, replacing the *l*_1_-penalty with an *l_q_*-penalty (0 <*q *<1) generalizes the lasso to bridge regression, which also has sparse and robust variants able to efficiently perform high-dimensional variable selection [24].

The presence of epistatic interactions, nonlinear effects, or non-independent observations may lower the performance of the regularized linear models. In such cases, performance may be enhanced by using lasso type models that allow for interactions between predictors and correlated observations [8,25], nonparameteric or semiparametric regularized regression models [26], or other procedures able to efficiently handle large numbers of interactions, such as random forests, or boosted regression trees [27].

## Conclusions

All the six models are additive and performed well for the simulated data set and may be expected to perform similarly well for traits where additive effects predominate and epistasis is less relevant.

## List of abbreviation used

ADAENET: Adaptive Elastic net; CV: Cross Validation; ENET: Elastic net; GS: Genomic Selection; GEBV: Genomic Estimated Breeding Value; GWAS: Genome-Wide Association Study; lasso: least absolute shrinkage and selection operator; RR: Ridge Regression; SNP: Single Nucleotide Polymorphisms; TBV: True Breeding Value; TGV: True Genomic Value.

## Competing interests

The authors declare that they have no competing interests.

## Authors' contributions

JOO conceived the study, conducted the statistical analysis and drafted the manuscript.

TSS participated in data preparation, analysis, and writing of the manuscript. HPP participated in discussions that helped improve the manuscript and oversaw the project. All the authors read and approved the manuscript.
